# Maximum Parsimony and the Skewness Test: A Simulation Study of the Limits of Applicability

**DOI:** 10.1371/journal.pone.0152656

**Published:** 2016-04-01

**Authors:** Jussi Määttä, Teemu Roos

**Affiliations:** Helsinki Institute for Information Technology HIIT, Department of Computer Science, University of Helsinki, Helsinki, Finland; University of Kansas, UNITED STATES

## Abstract

The maximum parsimony (MP) method for inferring phylogenies is widely used, but little is known about its limitations in non-asymptotic situations. This study employs large-scale computations with simulated phylogenetic data to estimate the probability that MP succeeds in finding the true phylogeny for up to twelve taxa and 256 characters. The set of candidate phylogenies are taken to be unrooted binary trees; for each simulated data set, the tree lengths of all (2*n* − 5)!! candidates are computed to evaluate quantities related to the performance of MP, such as the probability of finding the true phylogeny, the probability that the tree with the shortest length is unique, the probability that the true phylogeny has the shortest tree length, and the expected inverse of the number of trees sharing the shortest length. The tree length distributions are also used to evaluate and extend the skewness test of Hillis for distinguishing between random and phylogenetic data. The results indicate, for example, that the critical point after which MP achieves a success probability of at least 0.9 is roughly around 128 characters. The skewness test is found to perform well on simulated data and the study extends its scope to up to twelve taxa.

## Introduction

The maximum parsimony (MP) method for selecting a phylogenetic tree was developed in the early 1970’s (e.g. [1]). Its fundamental idea is to find the phylogeny that minimizes the amount of evolutionary change required (and thus maximizes *parsimony*). It is distinctly different from probabilistic methods, although it can be shown to be equivalent to the maximum likelihood approach for the exceedingly complex and unrealistic “no common mechanism” model [2]. While other methods have largely replaced MP, it remains in use due to its computational efficiency (pg. 269 [3]). Maximum parsimony has also been found to outperform likelihood methods when evolution is heterogeneous [4].

It is well-known that MP fails to be consistent under certain conditions [5, 6]. However, this is not a prohibitive restriction: sufficient conditions under which MP *is* consistent have also been derived (cf. [7] and the references therein), and one can easily find applied studies where MP performs well (e.g. [8]). Perhaps surprisingly, it appears that not much is known about the non-asymptotic boundaries within which MP fails or succeeds. This can be viewed as a distinction between studying *consistency*, i.e., the behavior of a method when the amount of data is “large enough,” and *efficiency*, or how much data a method requires in order to produce reliable results (note that we use the term *efficiency* in a broader sense than is often done in statistics where the efficiency of an unbiased estimator is inversely proportional to its variance [9]).

The goal of this paper is to study the efficiency of MP by employing large-scale computational experiments to find the boundaries that determine whether the reconstructions produced are trustworthy in the small sample regime where the behavior is not yet dominated by asymptotic effects. We generated a large amount of simulated phylogenetic data in various settings and computed the parsimony scores of *all* possible phylogenies. Our data sets included up to twelve taxa (corresponding to about 655 million candidate phylogenies) and up to 256 four-state characters. Our simulations allow us to give highly accurate estimates of when MP succeeds or fails.

Studying extremal inputs is important for developing an understanding of when a method is applicable and what are the limiting factors. Results regarding the consistency of MP provide at least partial answers to the question “what should I expect if I have lots of data?” Our study, on the other hand, attempts to answer the question “what should I expect if I have little data?”

We are not aware of any earlier work of this computational scale. Rice and Warnor [10] compared MP to two distance-based methods, with roughly the same number of simulated data sets, but they used networks of sixteen or more taxa, forcing them to resort to heuristic methods; their main finding was that MP was less affected by high mutation rates than the other two methods. A well-known simulation study [11] used only four taxa, but considered a wide range of methods and data-generating models. Another study [12] compared various phylogenetic algorithms, but there the focus was more on varying the data-generating models and on measuring the accuracies of the reconstructed phylogenies, whereas we fixed a single data-generating model and use a kind of binary metric where not finding the true phylogeny was treated as a complete failure. The consistency of maximum parsimony and other methods has also been examined empirically when the data model does not assume independence of the characters [13].

Our results also enabled us to measure the performance of the *skewness test* [14] that attempts to detect the presence of “phylogenetic signal” in a given data set by computing the third standardized moment of the distribution of the tree-length (parsimony) distribution. Hillis computed the critical values used by the test for up to eight taxa; we extend the scope of the skewness test to up to twelve taxa by providing the corresponding critical values for these more complex settings.

By phylogenetic signal, it is meant that the tree-length distribution induced by the data is significantly different from that typically produced by random data. Therefore, it should be emphasized that a positive result from the skewness test is not an indicator for or against the correctness of the tree reconstructed by MP; it simply means that the data are not likely to be random, warranting further study by reconstruction methods. The same applies to a whole range of methods and care should be taken in the correct interpretation of their results [15, 16]. While it may be argued that the presence of phylogenetic signal can be taken for granted with many biological data sets, it should be pointed out that reconstruction methods are also applied to other kinds of data (such as data from studies on cultural evolution [17]) for which it may not be obvious *a priori* that a phylogenetic signal is present.

One of the main motivations of this paper arises from the following observation where we compare the number of possible phylogenies and the number of possible parsimony scores that may be assigned to them. Suppose we have *m* characters on *n* observed taxa. We take all characters to have four possible states (A, C, G, T); hence, the data can be represented as an element of the set of all possible data sets $D^{\left(n,m\right)}={\left\{A,C,G,T\right\}}^{nm}$. Given a data set $D\in D^{\left(n,m\right)}$ and any unrooted binary tree $T\in T^{\left(n\right)}$ with *n* labeled leaves, we may compute *ℓ*(*T*; *D*), the *length* of the tree; this number tells the minimum number of changes that must have occurred in the process of evolution if we assume the phylogeny given by the tree. A tree $T^{*}\in T^{\left(n\right)}$ that minimizes *ℓ*(⋅; *D*) is called a *maximally parsimonious* tree. It is known that *ℓ*(*T*; *D*) is always an integer with 0 ≤ *ℓ*(*T*; *D*) ≤ (*n* − ⌈*n*/4⌉)*m* (e.g. [18]), so there are at most (*n* − ⌈*n*/4⌉)*m* + 1 possible lengths. On the other hand, the number of unrooted binary trees is $|T^{\left(n\right)}|=\left(2n-5\right)!!=1\cdot 3\cdot 5\cdot 7\cdot \ldots \cdot \left(2n-5\right)$ [19] which grows faster than exponentially as a function of *n*. Therefore, given the data, many different trees must have the same length; more precisely, at least

$$\begin{array}{c} \left\lceil \frac{\text{\# of trees}}{\text{\# of possible lengths}}\right\rceil \geq \left\lceil \frac{(2n-5)!!}{(n-\lceil n/4\rceil )m+1}\right\rceil \end{array}$$ (1)

trees will *always* have the same length.

It should be noted that under the null model, where data do not have phylogenetic structure, there are significant analytical results about the distributional properties of tree lengths [20]. We are not aware of any similar results for other models.

In any realistic setting, the value of Eq (1) will tend to infinity as *n* grows. In other words, as the number of taxa increases, it will become impossible to distinguish between a large number of possible trees by only looking at the tree lengths. This is not necessarily a serious problem, as the goal is principally only to find a tree with the minimal length. Furthermore, the lower bound given by Eq (1) for the number of trees sharing the same lengths becomes insignificant relative to $|T^{\left(n\right)}|$ when *n* is large, so quantifying the effect in more detail would require further theoretical results about how tight the lower bound is. But even if we disregard the lengths of all but the maximally parsimonious trees, there are few results regarding the conditions under which the shortest tree is unique: Steel and Penny (Prop. 9.4.2 in [18]) showed the somewhat weak requirement that *m* must grow at least logarithmically as a function of *n* (there also exists a more general result for binary characters, see Thm 14 in [21]), and it is known that the probability of the MP tree being unique tends to one as *m* tends to infinity if one considers two-state characters with random data [22]. Finally, one must of course consider the possibility that the true phylogeny may not always have the shortest tree length.

## Methods

### Generating trees

In order to generate all unrooted binary trees with a given number of leaves, we used the enumeration given by Rohlf [23] that bijectively maps the integers 1, 2, ⋯, (2*n* − 5)!! to the set of unrooted binary trees with *n* labeled leaves. The mapping is based on representing a tree as a tuple whose each element corresponds to the addition of a new node to the tree by splitting one of the edges; these tuples can be thought as mixed base numbers and they are representable as integers.

We used the Yule–Harding model [24] to generate random trees for our simulated data sets. Heard [25] points out that reconstructions from biological data tend to be more unbalanced than those predicted by the Yule–Harding model. However, we do not expect this to significantly bias our results, and the Yule–Harding model appears to a more reasonable choice than imposing a uniform distribution on $T^{\left(n\right)}$ would be [26].

### Generating simulated data

#### Phylogenetic data

For *m* characters on *n* taxa, we generated simulated data using the following procedure. First, we generated a random unrooted binary tree with *n* leaves using the Yule–Harding model. Then we arbitrarily rooted the tree and set all characters to random states on the root node (which corresponds to one of the leaves of the unrooted tree). We then proceeded down the tree using the Jukes–Cantor model [27], parametrized as follows. First, we fixed a mutation probability *q* ∈ (0, 0.75), which describes the probability of a character changing to a different state along an edge. Edge lengths were drawn from the exponential distribution with unit mean, so the corresponding substitution rate was *μ* = 4*q*/(3 − 4*q*). Strictly speaking, this procedure produces *extensions* of the *m* characters to the generated tree. For the final data set, we stored the character states of the root node and the *n* − 1 leaf nodes of the rooted tree, obtaining a phylogenetic data set corresponding to an unrooted binary tree with *n* leaves.

#### Random data

To calibrate the skewness test, we required data sets that do *not* have a phylogenetic structure. It has been a topic of debate how random data should be defined in phylogenetics: for instance, one might argue that data are random if they provide little (if any) information regarding tree choice [28]. In this article, we take random data to broadly mean any data that have been generated randomly without an underlying evolutionary or ancestral model. The way in which such random data are generated will always produce a bias in the results. For example, it is known that for two-state characters, if we generate each character independently and assign states to taxa uniformly at random, then the resulting distribution of trees reconstructed by maximum parsimony is uniform [22] if ties between multiple MP trees are resolved randomly; hence, the trees are less balanced on average than is usually desirable. In the same setting, if *n* = 6, any caterpillar tree is more likely to have the shortest length than a symmetrical tree at least when there are exactly two characters [29].

Consider a single four-state character on *n* taxa. If we were to assign all character states randomly, the probability of all states being the same would be much smaller than the probability that all states appear roughly the same number of times. In general, the length of a given tree depends only on the *patterns* of the characters. The possible patterns correspond to the elements of the set $P_{n}=\left\{\left(i,j,k,\ell \right)\in Z^{4}:0\leq i\leq j\leq k\leq \ell ,\;i+j+k+\ell =n\right\}$, where each (*i*, *j*, *k*, *ℓ*) may be interpreted, for instance, as *i* taxa having character state A and so on. (Obviously it makes no difference which element of (*i*, *j*, *k*, *ℓ*) is paired with which character state.)

We chose to generate random non-phylogenetic data as follows. For a given character, we first picked one pattern (*i*, *j*, *k*, *ℓ*) ∈ *P*_*n*_ uniformly at random. We then set the states of the first *i* taxa to A, the next *j* taxa to C, then *k* taxa to G and the rest to T, and finally shuffled the states in order to remove unwanted correlation between different characters. This procedure was repeated independently for each character. The purpose of this generation scheme was to produce a more varied collection of data sets.

### Experiments

We considered all combinations of (*n*, *m*, *q*) for *n* = 5, 6, ⋯, 12, *m* = 2, 4, 8, 16, ⋯, 256 and *q* = 0.08, 0.16, 0.24, ⋯, 0.48. For each of these settings, we generated *K* = 1000 simulated data sets (except for *n* = 12, for which we generated *K* = 500 data sets), and for each data set, we computed the lengths of all unrooted binary trees using the classic Fitch’s algorithm [1], which has a runtime of *O*(*nm*) and can be seen as a special case of a more general dynamic programming algorithm [3]. For every experiment, we stored the full distribution of tree lengths among the set of possible trees. We also separately recorded the lengths of the true tree and the maximally parsimonious tree.

It should be pointed out that instead of computing the lengths of all possible trees, one should usually use, for instance, a branch-and-bound algorithm [30] to find only the tree(s) with the shortest length. We needed all tree lengths to be able to study the skewness test.

All in all, the above experiments implied about 1.75 ⋅ 10^13^ executions of Fitch’s algorithm, so attention had to be paid to the practical issues regarding the computations. We used our own implementations of the necessary algorithms, implemented in highly optimized C++11. The *SWIG* library was used to interface the C++11 program code from Python in order to enable us to use the *Apache Spark* engine for distributed computing. The software is provided in S1 Software Package. The experiments were distributed among 100 cluster nodes; each node was equipped with two four-core Intel Xeon E5540 processors (with each core capable of running two threads simultaneously due to hyper-threading), allowing us to run sixteen simultaneous experiments on each node. With this setup, running all the experiments required approximately 279 hours of wall-clock time.

### Quantities derived from the experiments

#### Probability of success and related quantities

For every setting characterized by the triplet (*n*, *m*, *q*), we wanted to estimate the probability that the maximum parsimony method was successful in finding the true phylogeny. To describe how we estimated this probability, we first need to establish further notation. Consider the generating model described above. Let $T^{*}\in T^{\left(n\right)}$ be the true phylogeny and let the set $\hat{T}\subseteq T^{\left(n\right)}$ contain all trees that share the shortest length for the generated data; the number of elements in this set is denoted by $|\hat{T}|$. The random variable $\tilde{T}$ picks one tree from $\hat{T}$, assigning uniform probabilities to all trees; a similar approach was used by Zhu and Steel [22].

We say that the MP method succeeds if the true phylogeny has the shortest length and, if there are multiple phylogenies sharing the same shortest length, the one picked by $\tilde{T}$ is the true phylogeny T*. This may be expressed as

$$\begin{array}{ccc} \mathrm{Pr}[\text{success}] & = & \mathrm{Pr}\left[T^{*}=\tilde{T}\right]=\mathrm{Pr}\left[T^{*}=\tilde{T}\wedge T^{*}\in \hat{T}\right]\\ & = & \sum_{k=1}^{(2n-5)!!}\mathrm{Pr}[T^{*}=\tilde{T}\wedge T^{*}\in \hat{T}\wedge |\hat{T}\left|=k\right]\\ & = & \sum_{k=1}^{(2n-5)!!}\mathrm{Pr}[T^{*}=\tilde{T}|T^{*}\in \hat{T}\wedge |\hat{T}|=k]\mathrm{Pr}[T^{*}\in \hat{T}\wedge |\hat{T}\left|=k\right]\\ & = & \sum_{k=1}^{(2n-5)!!}\frac{1}{k}\mathrm{Pr}\left[\frac{\chi (T^{*}\in \hat{T})}{|\hat{T}|}=\frac{1}{k}\right]\\ & = & E[\frac{\chi (T^{*}\in \hat{T})}{|\hat{T}|}] \end{array}$$ (2)

where $\chi \left(T^{*}\in \hat{T}\right)\in \left\{0,1\right\}$ is an indicator variable and the probabilities and expectations are over the simulated data (T*, *D*) and the estimator $\tilde{T}$. Suppose then that we perform *K* experiments with simulated data, indexed by *i* = 1, 2, ⋯, *K*. The law of large numbers allows us to approximate the probability of success with the following formula:

$$\begin{array}{c} \mathrm{Pr}\left[\text{success}\right]\approx \frac{1}{K}\sum_{i=1}^{K}\frac{\chi (T_{i}^{*}\in {\hat{T}}_{i})}{|{\hat{T}}_{i}|}\text{.} \end{array}$$ (3)

Similarly, one may estimate the probability that the true phylogeny has the shortest tree length:

$$\begin{array}{c} \mathrm{Pr}\left[T^{*}\in \hat{T}\right]\approx \frac{1}{K}\sum_{i=1}^{K}\chi \left(T_{i}^{*}\in {\hat{T}}_{i}\right)\text{,} \end{array}$$ (4)

and the expected inverse of the number of trees sharing the shortest length:

$$\begin{array}{c} E\left[\frac{1}{|\hat{T}|}\right]\approx \frac{1}{K}\sum_{i=1}^{K}\frac{1}{|{\hat{T}}_{i}|}\text{.} \end{array}$$ (5)

(Note that Pr[success], as given by Eq (3), is not, in general, the same as the product of Eqs (4) and (5).) Also of interest is the probability that the tree with the shortest length is unique:

$$\begin{array}{c} \mathrm{Pr}[|\hat{T}|=1]\approx \frac{1}{K}\sum_{i=1}^{K}\chi (|{\hat{T}}_{i}\left|=1\right)\text{.} \end{array}$$ (6)

We omitted the study of the probability of the event that the true phylogeny is the unique tree with the shortest length, i.e., $\mathrm{Pr}[T^{*}\in \hat{T}\wedge |\hat{T}|=1]$, since it is not unusual for MP to give multiple solutions and treating this as a complete failure would be unreasonable. We also limited the scope of our paper by excluding the use of tree distance metrics for more fine-grained quantification of the level of success or failure.

#### Skewness of the tree length distribution

Fitch [31] was one of the first authors to suggest examining the distribution of all tree lengths for a given data set. This idea was developed further by Hillis [14], who proposed the *skewness test* for estimating whether a given data set is random or potentially has some phylogenetic structure. More specifically, he considered the skewness of the tree length distribution, here defined as the third standardized moment

$$\begin{array}{c} \gamma_{1}=\frac{\sum_{T\in T^{\left(n\right)}}{\left(\ell (T;D)-\mu \right)}^{3}}{|T^{\left(n\right)}|\;\sigma^{3}} \end{array}$$ (7)

where *μ* and *σ* are the mean and the standard deviation of *ℓ*(*T*; *D*) over all $T\in T^{\left(n\right)}$. The statistic *γ*_1_ is zero when the distribution is symmetrical; distributions with *γ*_1_ < 0 are said to be left-skewed and those with *γ*_1_ > 0 are right-skewed. Hillis found that random data (with no underlying phylogenetic structure) produced distributions with a small negative skewness, whereas data with a real phylogenetic “signal” produced a skewness that was significantly more negative. This allowed him to define a statistical test for the presence of significant phylogenetic signal. The approach was further examined by Huelsenbeck [32].

The skewness test of Hillis is constructed from the distribution of the *γ*_1_ statistic, computed for a large number of fully random data sets. One takes e.g. the 1st or the 5th percentile of this distribution as a critical value. The test is applied to a data set by computing the skewness of the tree length distribution induced by the data; if the skewness is smaller than the critical value, then according to the test, the data are not likely to be random and hence may well contain some phylogenetic signal.

Källersjö et al. [33] criticized the skewness test. In their paper, they demonstrated simple data sets on which the approach fails. These data sets might be called pathological, and it is disputable how much they influence the average performance of the skewness test. Perhaps more importantly, they pointed out that the skewness test is affected by the relative frequencies of character states and also when different branches may have different mutation probabilities. Consequently, the validity of the skewness test is presently unknown in all but the simplest settings.

Importantly, the practicality of the skewness test is limited by the need to compute the lengths of all trees; naïve random sampling of the trees is not a solution because of the long and extremely thin tails typical to the distributions in question. Le Quesne [34] did use a stochastic approach for estimating the skewness for up to 23 taxa, but the bias introduced by his method is unknown, as is the accuracy of his results, given that he sampled only 10240 out of (2 ⋅ 23 − 5)!! ≈ 1.3 ⋅ 10^25^ possible trees. Hence, it is not surprising that the literature on the topic [14, 31, 32] only considers cases where the number of taxa is at most eight (except for the two counterexample data sets given in [33]). However, it is conceivable that there might exist a sampling strategy for trees that would provide a good approximation of the skewness of the tree length distribution for large number of taxa; we leave this as an open question.

In this work, we investigated the performance of the skewness test within all our test settings, up to twelve taxa and 256 characters. For each (*n*, *m*), *n* = 5, 6, ⋯, 12, *m* = 2, 4, 8, 16, ⋯, 256, we generated 3000 instances of random non-phylogenetic data as discussed in the Methods section. These computations took an additional 421 hours of wall-clock time on our cluster setup. By computing the distribution of the skewness statistics for each of these settings for random data, we were able to calculate critical values similar to those given in Table 13-1 of [14]. The skewness test could then be applied to our simulated data that we knew to have a phylogenetic structure. The results of this experiment will make it clear if the skewness test is also applicable in cases where the number of taxa lies between nine and twelve. Furthermore, in our simulated data, as the mutation probability *q* increases, finding the true phylogeny becomes more difficult; hence, it seems reasonable to hypothesize that for high values of *q* (e.g., *q* = 0.48), the skewness test will fail more often than when *q* is small (e.g., *q* = 0.08).

## Results

In addition to the analysis below, we provide the results of our simulations in full (see S1 Table).

### Probabilities

**Fig 1:**
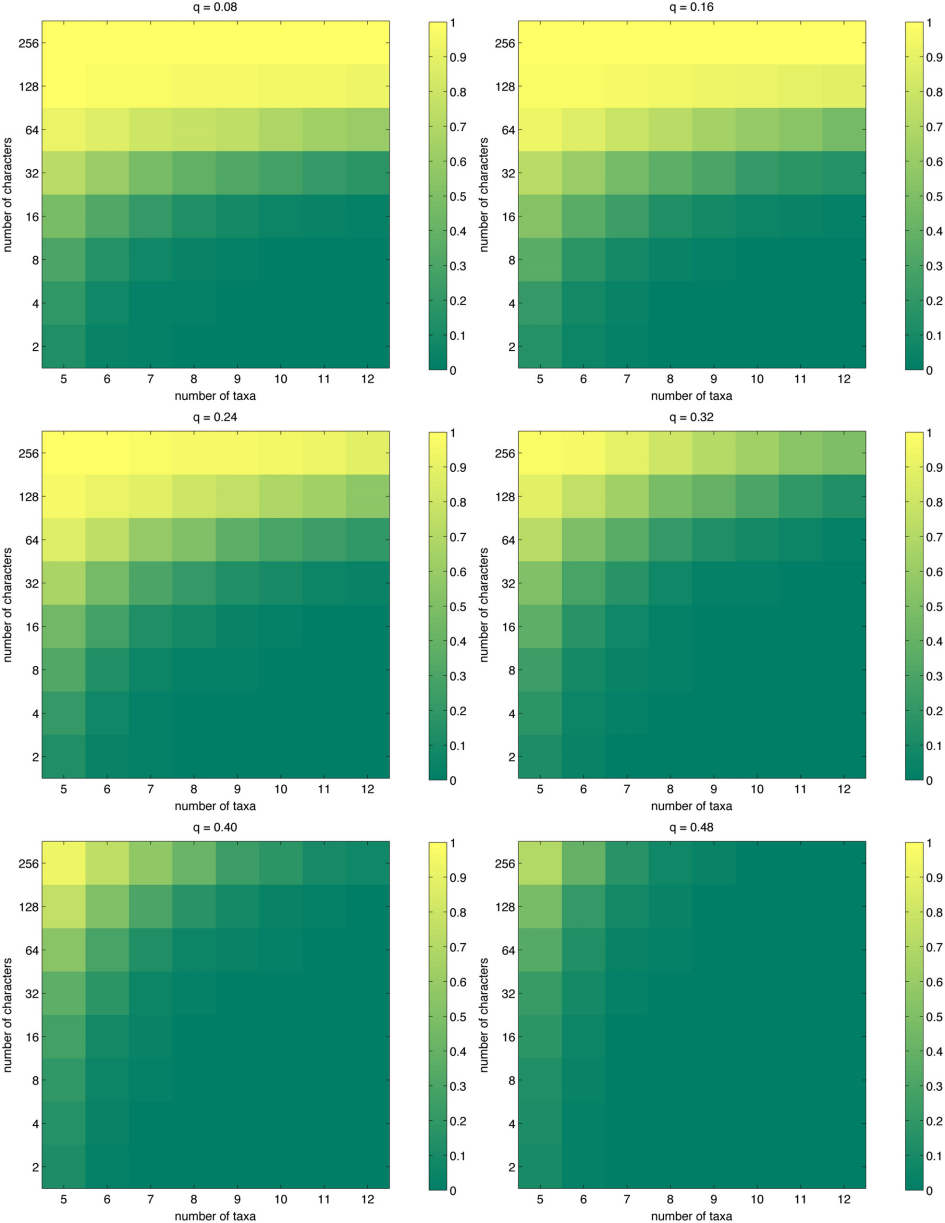
The probability of success for simulated phylogenetic data, as approximated by Eq (3).

Fig 1 displays the success probability, separately visualized for each of the mutation probabilities *q* = 0.08, 0.16, ⋯, 0.48. The same information is given numerically in Table 1, where success probabilities of at least 0.90 are highlighted. In the most amenable setting (*q* = 0.08), having 64 characters is not enough to obtain a success probability of 0.90 except for the case *n* = 5; for 6 ≤ *n* ≤ 12, having 128 characters is sufficient. The extreme case *q* = 0.48 is intractable; even for five taxa and 256 characters, the true phylogeny could be inferred in only about 70% of the experiments. Fig 1 clearly shows that if the number of characters is kept fixed, the probability of success rapidly decreases as the number of taxa increases.

**Table 1:** The probability of success for all settings (*n*, *m*, *q*), as approximated by Eq (3). Probabilities of at least 0.90 are shown in boldface.

*n*	m	q = 0.08	q = 0.16	q = 0.24	q = 0.32	q = 0.40	q = 0.48
5	2	0.13	0.15	0.13	0.12	0.11	0.09
5	4	0.19	0.20	0.21	0.18	0.14	0.11
5	8	0.31	0.35	0.31	0.23	0.19	0.13
5	16	0.48	0.53	0.45	0.37	0.28	0.18
5	32	0.74	0.74	0.67	0.51	0.36	0.23
5	64	**0.92**	**0.93**	0.87	0.74	0.54	0.33
5	128	**0.99**	**0.99**	**0.98**	0.89	0.76	0.48
5	256	**1.00**	**1.00**	**1.00**	**0.99**	**0.93**	0.70
6	2	0.03	0.04	0.03	0.03	0.02	0.02
6	4	0.06	0.08	0.06	0.05	0.03	0.02
6	8	0.14	0.18	0.13	0.08	0.05	0.03
6	16	0.31	0.34	0.27	0.15	0.08	0.04
6	32	0.60	0.61	0.45	0.28	0.18	0.08
6	64	0.87	0.87	0.74	0.50	0.28	0.12
6	128	**0.98**	**0.99**	**0.93**	0.76	0.51	0.21
6	256	**1.00**	**1.00**	**0.99**	**0.96**	0.75	0.39
7	2	0.01	0.01	0.01	0.01	0.00	0.00
7	4	0.02	0.03	0.02	0.01	0.00	0.00
7	8	0.07	0.08	0.05	0.03	0.02	0.00
7	16	0.21	0.23	0.13	0.06	0.03	0.01
7	32	0.48	0.45	0.31	0.15	0.05	0.02
7	64	0.81	0.80	0.59	0.35	0.13	0.03
7	128	**0.98**	**0.97**	0.89	0.64	0.30	0.08
7	256	**1.00**	**1.00**	**0.99**	**0.90**	0.57	0.16
8	2	0.00	0.00	0.00	0.00	0.00	0.00
8	4	0.01	0.01	0.01	0.00	0.00	0.00
8	8	0.03	0.03	0.02	0.01	0.00	0.00
8	16	0.13	0.13	0.08	0.02	0.01	0.00
8	32	0.39	0.36	0.21	0.06	0.02	0.00
8	64	0.78	0.73	0.51	0.21	0.05	0.01
8	128	**0.97**	**0.95**	0.82	0.48	0.15	0.03
8	256	**1.00**	**1.00**	**0.97**	0.80	0.41	0.07
9	2	0.00	0.00	0.00	0.00	0.00	0.00
9	4	0.00	0.00	0.00	0.00	0.00	0.00
9	8	0.01	0.02	0.01	0.00	0.00	0.00
9	16	0.08	0.07	0.03	0.01	0.00	0.00
9	32	0.32	0.30	0.12	0.02	0.00	0.00
9	64	0.74	0.66	0.37	0.12	0.02	0.00
9	128	**0.97**	**0.94**	0.77	0.40	0.08	0.01
9	256	**1.00**	**1.00**	**0.97**	0.72	0.24	0.03
10	2	0.00	0.00	0.00	0.00	0.00	0.00
10	4	0.00	0.00	0.00	0.00	0.00	0.00
10	8	0.01	0.01	0.00	0.00	0.00	0.00
10	16	0.05	0.05	0.01	0.00	0.00	0.00
10	32	0.27	0.20	0.10	0.01	0.00	0.00
10	64	0.69	0.59	0.28	0.07	0.01	0.00
10	128	**0.96**	**0.92**	0.69	0.30	0.03	0.00
10	256	**1.00**	**1.00**	**0.95**	0.64	0.17	0.01
11	2	0.00	0.00	0.00	0.00	0.00	0.00
11	4	0.00	0.00	0.00	0.00	0.00	0.00
11	8	0.00	0.00	0.00	0.00	0.00	0.00
11	16	0.03	0.03	0.01	0.00	0.00	0.00
11	32	0.20	0.18	0.05	0.00	0.00	0.00
11	64	0.64	0.55	0.24	0.05	0.00	0.00
11	128	**0.95**	**0.91**	0.64	0.19	0.02	0.00
11	256	**1.00**	**1.00**	**0.93**	0.55	0.10	0.00
12	2	0.00	0.00	0.00	0.00	0.00	0.00
12	4	0.00	0.00	0.00	0.00	0.00	0.00
12	8	0.00	0.00	0.00	0.00	0.00	0.00
12	16	0.02	0.01	0.00	0.00	0.00	0.00
12	32	0.18	0.14	0.03	0.00	0.00	0.00
12	64	0.60	0.46	0.19	0.02	0.00	0.00
12	128	**0.93**	0.89	0.55	0.13	0.00	0.00
12	256	**1.00**	**0.99**	0.88	0.50	0.07	0.00

Looking at Fig 1 and noting the logarithmic scale in the vertical axis, it appears that if one wants to keep the success probability at some fixed level, the rate at which one has to increase the number of characters when the number of taxa is increased seems to roughly match (if not exceed) the logarithmic lower bound given in [18]. Our data are not suitable for quantifying this relationship more specifically; it would require varying the number of characters on a scale denser than the logarithmic one we have used to bring out interesting behavior while not exceeding available computing time.

**Fig 2:**
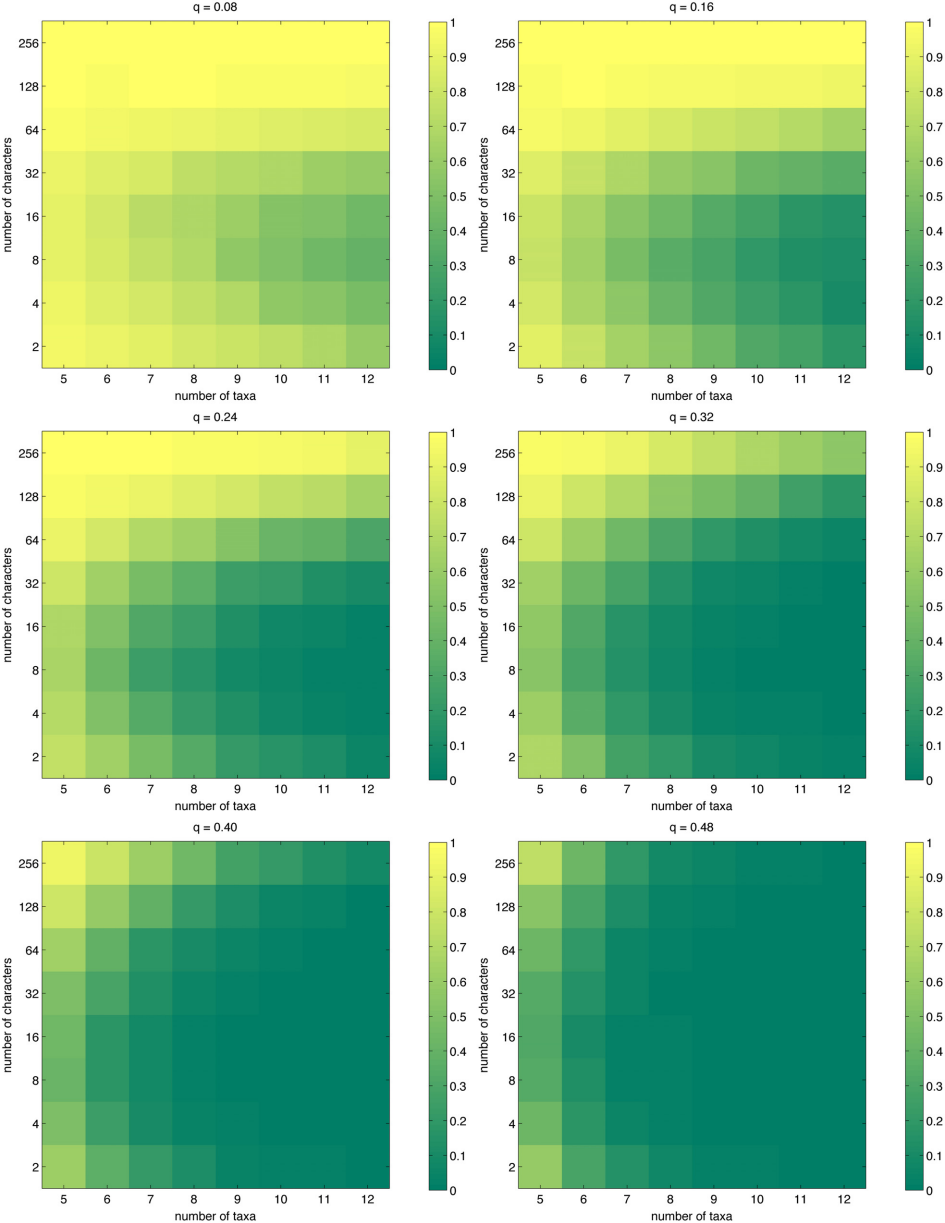
The probability that the true phylogeny has the shortest tree length, as approximated by Eq (4).

As mentioned in the Introduction, it is not clear how often the true phylogeny actually has the shortest tree length. Fig 2 visualizes the probability of this happening, as approximated by Eq (4). Perhaps the most interesting feature in the figure is the behavior along the vertical axes. Except for intractable settings, if one considers some fixed number of taxa, the true phylogeny is more likely to have the shortest length not only when there are sufficiently many characters, but also when the number of characters is extremely small. Between these extrema, the probability is at its lowest. Consider, for instance, the case *n* = 12 for *q* = 0.08: there having only two characters gives a noticeably higher probability than when there are eight characters. The explanation for this phenomenon is that when the number of characters is extremely small, the number of possible tree lengths becomes so small that the true phylogeny is more likely to have the shortest length by chance (that is, not because of some discernible phylogenetic signal in the data). It is apparent that this quantity is not a particularly good measure of phylogeny reconstruction performance.

**Fig 3:**
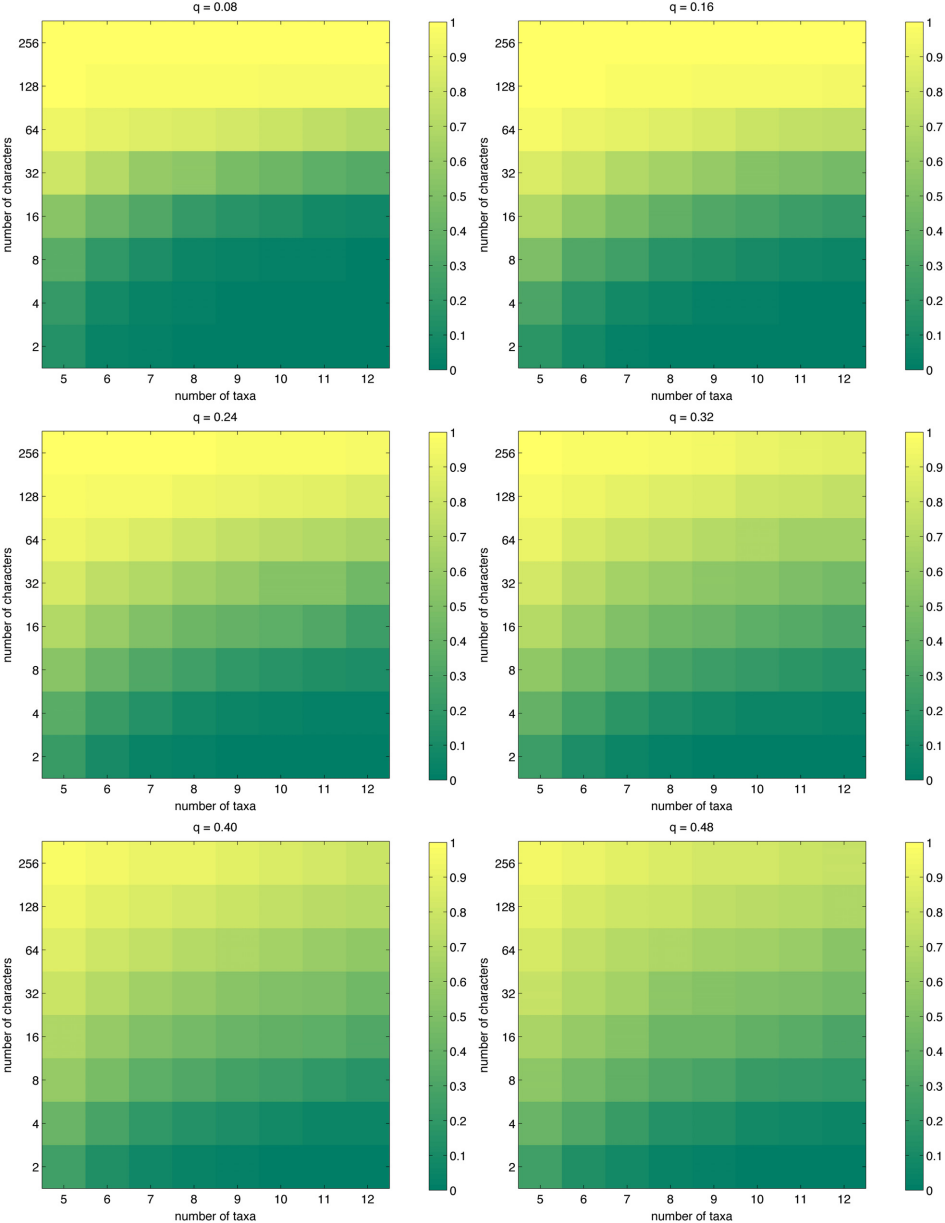
The expectation $E[1/|\hat{T}|]$, i.e., the expected inverse of the number of trees sharing the shortest length, as approximated by Eq (5).

**Fig 4:**
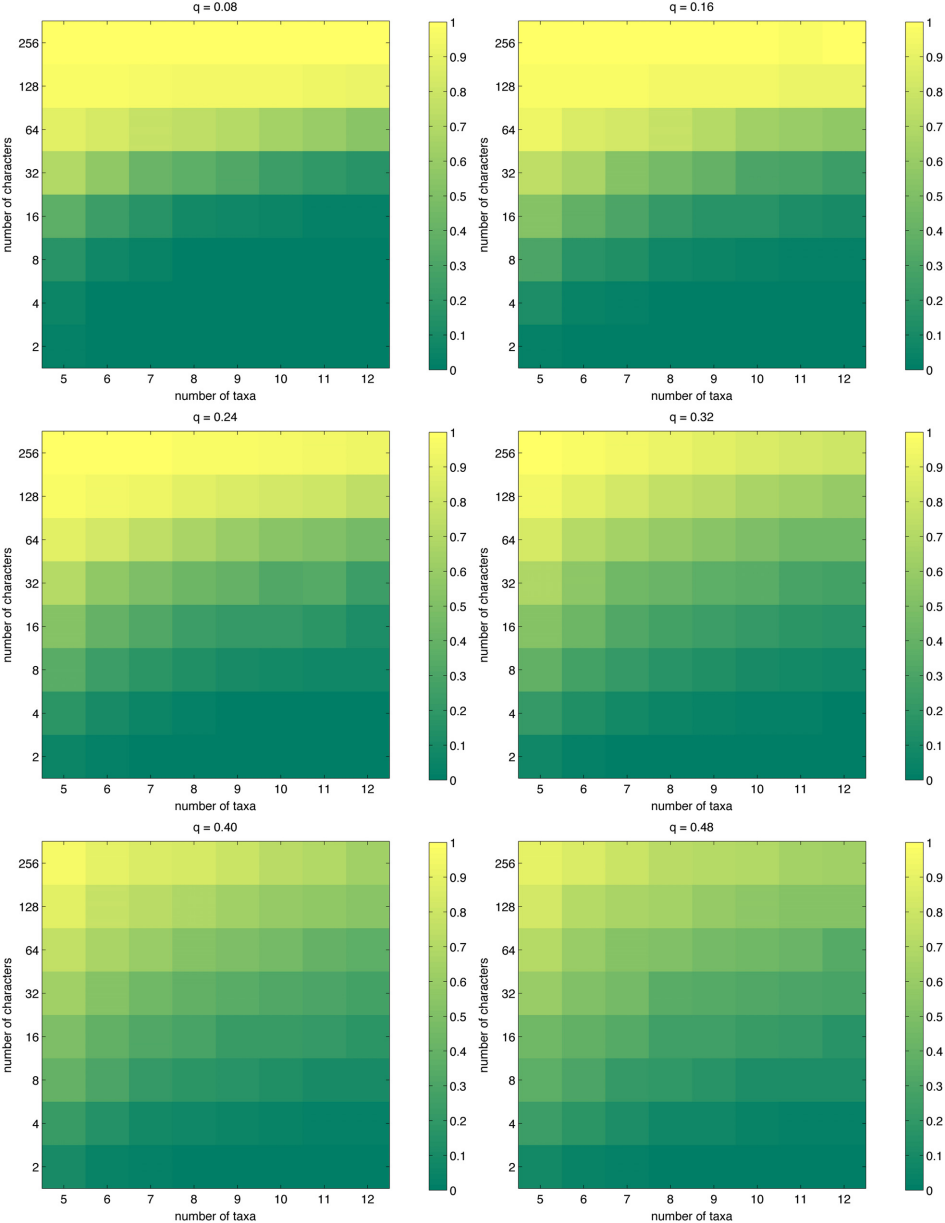
The probability that the tree with the shortest length is unique, as approximated by Eq (6).

Maximum parsimony may often find multiple trees that share the shortest length, which presents an additional conundrum to the interpretation of the results. Additionally, when the number of taxa is so large that even branch-and-bound search [30] becomes unfeasible and one must switch to heuristic search, and even if one were to assume that a global optimum is found, it is not possible to know whether the solution is unique. We measured the expected inverse of the number of trees sharing the shortest length (Eq (5)). The results, shown in Fig 3, are surprisingly good: in all settings considered, having 64 characters easily keeps the number of trees in the single digits. It is also interesting that for low mutation probabilities, the transition from uniqueness to multiple shortest trees is sharper than for high mutation probabilities, where it is perhaps the case that there is more diversity in the data, aiding MP in differentiating between trees. In fact, for e.g. *n* = 12, *m* = 32, the mutation probability *q* = 0.48 is more favorable than *q* = 0.08 from this point of view. We also computed the probability that the shortest length is exclusive to a single tree (Eq (6)); our results on this closely related quantity are shown in Fig 4, which is qualitatively similar to Fig 3.

### Skewness

**Fig 5:**
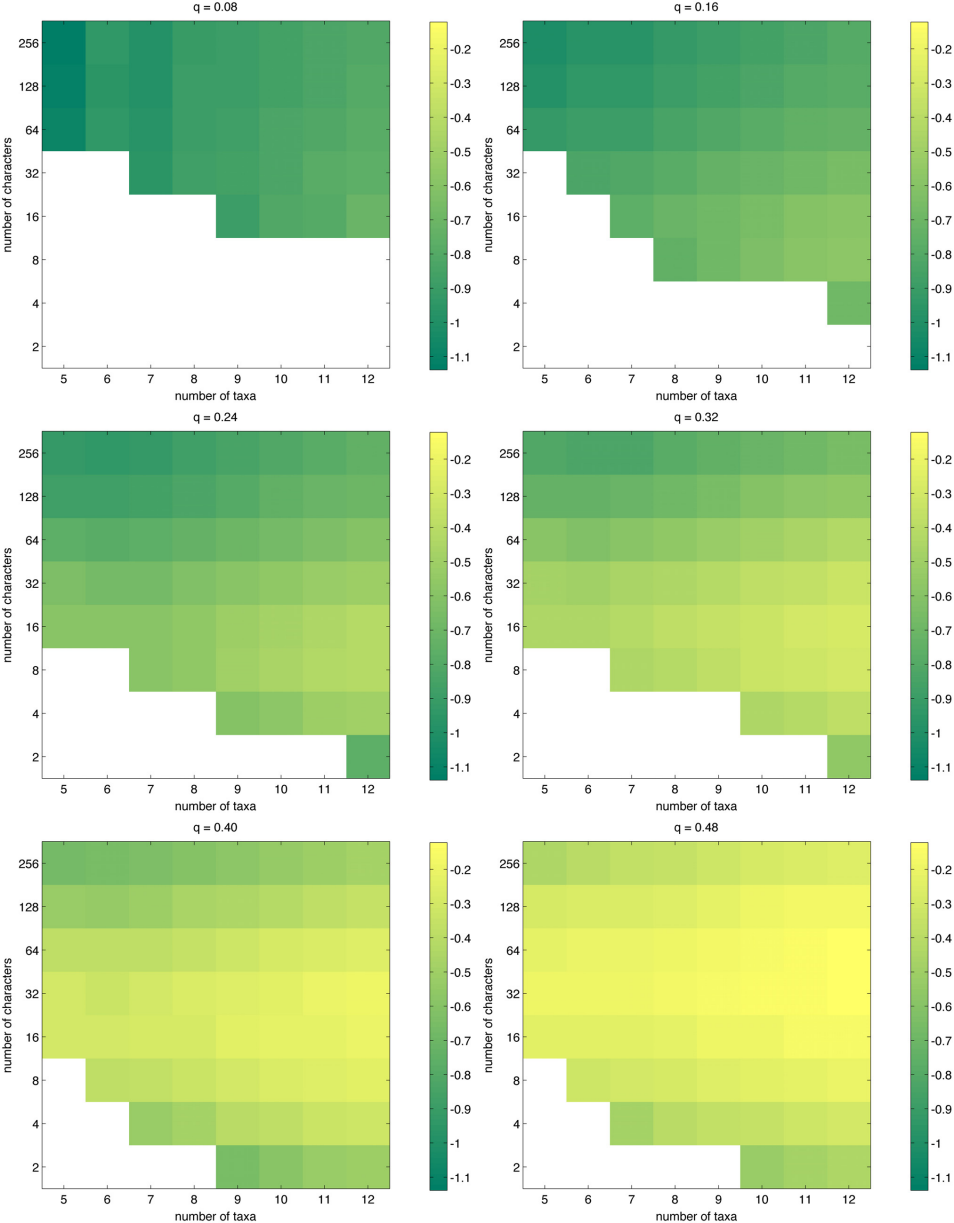
Approximate value of E[*γ*_1_∣*σ* > 0]. Values are not shown for settings where at least one trial produced a degenerate skewness distribution (*σ* = 0); these cases are indicated by the white blocks at the bottom left regions of the figures.

Skewness, as per Eq (7), is only defined when the tree length distribution considered has a strictly positive variance. However, in many of the cases (*n*, *m*, *q*) that we considered, some of the simulated data sets had every tree share the same length (and hence *σ* = 0). While such data sets could be rejected in the generation step, this would result in a bias that would show parsimony in an overly positive light; after all, other methods may well cope with such data sets even when parsimony does not. Despite the occurrence of these degenerate distributions, we would like to visualize the effect of the parameters *n*, *m* and *q* on the expected skewness of the tree length distributions. Therefore, we show approximate values for E[*γ*_1_∣*σ* > 0] in Fig 5. For triplets (*n*, *m*, *q*) where we encountered at least one degenerate distribution, we do not display these expectations; the reason for this omission is that the fraction of cases where *σ* = 0 varies between settings and it is not clear to us whether it makes sense to compare these values when the probability of getting a degenerate distribution is non-negligible. In any case, as can be seen from the figure, the degenerate cases occur mostly in situations where there is little hope of inferring the true phylogeny.

Our main results related to skewness are shown in Tables 2 and 3, where we show critical values for the skewness test for various settings (*n*, *m*) and for two confidence levels (95% and 99%). In some of the more difficult settings, such as *n* = 6, *m* = 4, some of the random data sets used for deriving the critical values produced degenerate tree length distributions; therefore, we slightly redefined the skewness test by adding the additional condition that the test treats degenerate tree length distributions as containing no phylogenetic signal. The critical values were then computed from the fraction of random data sets that exhibit a non-degenerate distribution; and when applying the test to simulated phylogenetic data, if such data produce a degenerate distribution, we say that the skewness test fails to detect the phylogenetic signal in the data.

**Table 2:** Critical values for the skewness test (95% confidence limit), and the rates at which the test correctly detects phylogenetic signal in our simulated data for various values of the mutation probability *q*. Correctness rates of at least 0.90 are shown in boldface.

			Correctness rate					
*n*	*m*	Critical value	*q* = 0.08	*q* = 0.16	*q* = 0.24	*q* = 0.32	*q* = 0.40	*q* = 0.48
5	2	−1.50	0.00	0.00	0.00	0.00	0.00	0.00
5	4	−1.50	0.00	0.00	0.00	0.00	0.00	0.00
5	8	−1.50	0.00	0.00	0.00	0.00	0.00	0.00
5	16	−1.34	0.32	0.08	0.02	0.01	0.00	0.00
5	32	−1.19	0.42	0.12	0.04	0.02	0.01	0.00
5	64	−1.12	0.55	0.19	0.09	0.03	0.01	0.01
5	128	−1.14	0.46	0.15	0.04	0.02	0.01	0.00
5	256	−1.16	0.43	0.08	0.02	0.01	0.00	0.00
6	2	−2.04	0.00	0.00	0.00	0.00	0.00	0.00
6	4	−2.04	0.00	0.00	0.00	0.01	0.01	0.00
6	8	−1.36	0.40	0.18	0.05	0.01	0.00	0.00
6	16	−0.85	0.52	0.40	0.21	0.10	0.05	0.02
6	32	−0.80	0.61	0.55	0.33	0.15	0.06	0.02
6	64	−0.76	0.70	0.75	0.58	0.29	0.10	0.03
6	128	−0.66	**0.90**	**0.97**	**0.94**	0.67	0.34	0.08
6	256	−0.62	**0.97**	**1.00**	**1.00**	**0.94**	0.59	0.20
7	2	−2.47	0.00	0.00	0.00	0.00	0.00	0.00
7	4	−2.47	0.00	0.00	0.00	0.00	0.00	0.00
7	8	−1.19	0.35	0.14	0.04	0.01	0.01	0.00
7	16	−0.79	0.67	0.43	0.21	0.08	0.02	0.02
7	32	−0.66	**0.91**	0.79	0.53	0.21	0.06	0.02
7	64	−0.61	**0.99**	**0.98**	0.85	0.50	0.15	0.04
7	128	−0.57	**1.00**	**1.00**	**1.00**	0.88	0.39	0.08
7	256	−0.56	**1.00**	**1.00**	**1.00**	**0.99**	0.72	0.17
8	2	−2.85	0.00	0.00	0.00	0.00	0.00	0.00
8	4	−2.47	0.16	0.05	0.02	0.00	0.00	0.00
8	8	−0.87	0.52	0.29	0.11	0.03	0.01	0.01
8	16	−0.62	0.81	0.65	0.38	0.14	0.05	0.01
8	32	−0.53	**0.94**	**0.92**	0.72	0.34	0.09	0.03
8	64	−0.49	**1.00**	**1.00**	**0.96**	0.69	0.21	0.04
8	128	−0.49	**1.00**	**1.00**	**1.00**	**0.95**	0.48	0.09
8	256	−0.44	**1.00**	**1.00**	**1.00**	**1.00**	**0.92**	0.31
9	2	−3.18	0.00	0.00	0.00	0.00	0.00	0.00
9	4	−2.09	0.20	0.03	0.01	0.00	0.00	0.00
9	8	−0.88	0.45	0.18	0.04	0.01	0.00	0.00
9	16	−0.58	0.88	0.69	0.34	0.11	0.03	0.01
9	32	−0.50	**0.99**	**0.97**	0.73	0.27	0.06	0.01
9	64	−0.45	**1.00**	**1.00**	**0.99**	0.72	0.18	0.02
9	128	−0.39	**1.00**	**1.00**	**1.00**	**0.99**	0.66	0.12
9	256	−0.36	**1.00**	**1.00**	**1.00**	**1.00**	**0.97**	0.40
10	2	−3.47	0.05	0.01	0.00	0.00	0.00	0.00
10	4	−1.63	0.22	0.07	0.01	0.00	0.00	0.00
10	8	−0.69	0.64	0.35	0.08	0.02	0.00	0.00
10	16	−0.50	**0.92**	0.82	0.41	0.12	0.02	0.01
10	32	−0.41	**1.00**	**0.99**	0.88	0.44	0.09	0.02
10	64	−0.36	**1.00**	**1.00**	**1.00**	0.85	0.30	0.04
10	128	−0.33	**1.00**	**1.00**	**1.00**	**1.00**	0.74	0.13
10	256	−0.33	**1.00**	**1.00**	**1.00**	**1.00**	**0.98**	0.39
11	2	−3.75	0.00	0.00	0.00	0.00	0.00	0.00
11	4	−1.61	0.23	0.05	0.01	0.00	0.00	0.00
11	8	−0.72	0.54	0.22	0.04	0.01	0.00	0.00
11	16	−0.46	**0.96**	0.88	0.44	0.11	0.03	0.00
11	32	−0.39	**1.00**	**1.00**	**0.91**	0.40	0.06	0.01
11	64	−0.32	**1.00**	**1.00**	**1.00**	**0.92**	0.31	0.04
11	128	−0.29	**1.00**	**1.00**	**1.00**	**1.00**	0.79	0.14
11	256	−0.27	**1.00**	**1.00**	**1.00**	**1.00**	**0.99**	0.46
12	2	−3.75	0.06	0.02	0.00	0.00	0.00	0.00
12	4	−1.58	0.22	0.03	0.00	0.00	0.00	0.00
12	8	−0.58	0.75	0.43	0.12	0.02	0.00	0.00
12	16	−0.40	**0.99**	**0.94**	0.56	0.13	0.02	0.00
12	32	−0.33	**1.00**	**1.00**	**0.96**	0.49	0.08	0.01
12	64	−0.28	**1.00**	**1.00**	**1.00**	**0.95**	0.40	0.05
12	128	−0.25	**1.00**	**1.00**	**1.00**	**1.00**	0.86	0.17
12	256	−0.24	**1.00**	**1.00**	**1.00**	**1.00**	**1.00**	0.52

**Table 3:** Critical values for the skewness test (99% confidence limit), and the rates at which the test correctly detects phylogenetic signal in our simulated data for various values of the mutation probability *q*. Correctness rates of at least 0.90 are shown in boldface.

			Correctness rate					
*n*	*m*	Critical value	*q* = 0.08	*q* = 0.16	*q* = 0.24	*q* = 0.32	*q* = 0.40	*q* = 0.48
5	2	−1.50	0.00	0.00	0.00	0.00	0.00	0.00
5	4	−1.50	0.00	0.00	0.00	0.00	0.00	0.00
5	8	−1.50	0.00	0.00	0.00	0.00	0.00	0.00
5	16	−1.33	0.38	0.09	0.02	0.01	0.00	0.00
5	32	−1.19	0.42	0.13	0.04	0.02	0.01	0.00
5	64	−1.11	0.55	0.20	0.09	0.03	0.01	0.01
5	128	−1.14	0.48	0.16	0.05	0.02	0.01	0.01
5	256	−1.15	0.46	0.10	0.03	0.01	0.01	0.00
6	2	−2.04	0.00	0.00	0.00	0.00	0.00	0.00
6	4	−2.04	0.00	0.00	0.00	0.01	0.01	0.00
6	8	−1.24	0.41	0.21	0.07	0.02	0.01	0.00
6	16	−0.84	0.52	0.40	0.23	0.10	0.05	0.02
6	32	−0.80	0.61	0.56	0.33	0.15	0.06	0.02
6	64	−0.76	0.71	0.76	0.59	0.30	0.10	0.03
6	128	−0.66	**0.90**	**0.98**	**0.94**	0.68	0.34	0.08
6	256	−0.61	**0.98**	**1.00**	**1.00**	**0.95**	0.61	0.21
7	2	−2.47	0.00	0.00	0.00	0.00	0.00	0.00
7	4	−2.47	0.00	0.00	0.00	0.00	0.00	0.00
7	8	−1.17	0.35	0.15	0.05	0.01	0.01	0.00
7	16	−0.78	0.68	0.44	0.22	0.09	0.02	0.02
7	32	−0.66	**0.91**	0.79	0.54	0.22	0.06	0.02
7	64	−0.60	**1.00**	**0.98**	0.85	0.50	0.15	0.04
7	128	−0.57	**1.00**	**1.00**	**1.00**	0.88	0.40	0.08
7	256	−0.56	**1.00**	**1.00**	**1.00**	**0.99**	0.73	0.18
8	2	−2.85	0.00	0.00	0.00	0.00	0.00	0.00
8	4	−2.08	0.19	0.06	0.02	0.00	0.00	0.00
8	8	−0.86	0.53	0.30	0.12	0.03	0.01	0.01
8	16	−0.61	0.81	0.66	0.38	0.14	0.05	0.01
8	32	−0.52	**0.95**	**0.93**	0.73	0.36	0.10	0.04
8	64	−0.49	**1.00**	**1.00**	**0.96**	0.69	0.21	0.04
8	128	−0.49	**1.00**	**1.00**	**1.00**	**0.96**	0.48	0.10
8	256	−0.44	**1.00**	**1.00**	**1.00**	**1.00**	**0.92**	0.31
9	2	−3.18	0.00	0.00	0.00	0.00	0.00	0.00
9	4	−2.04	0.20	0.04	0.01	0.00	0.00	0.00
9	8	−0.88	0.45	0.19	0.04	0.01	0.00	0.00
9	16	−0.58	0.88	0.70	0.34	0.11	0.03	0.01
9	32	−0.50	**0.99**	**0.97**	0.73	0.27	0.06	0.01
9	64	−0.45	**1.00**	**1.00**	**0.99**	0.73	0.20	0.03
9	128	−0.39	**1.00**	**1.00**	**1.00**	**0.99**	0.67	0.13
9	256	−0.36	**1.00**	**1.00**	**1.00**	**1.00**	**0.97**	0.40
10	2	−3.47	0.05	0.01	0.00	0.00	0.00	0.00
10	4	−1.62	0.27	0.07	0.02	0.00	0.00	0.00
10	8	−0.69	0.64	0.35	0.08	0.02	0.00	0.00
10	16	−0.50	**0.92**	0.82	0.41	0.12	0.02	0.01
10	32	−0.41	**1.00**	**0.99**	0.88	0.45	0.10	0.02
10	64	−0.36	**1.00**	**1.00**	**1.00**	0.85	0.30	0.04
10	128	−0.33	**1.00**	**1.00**	**1.00**	**1.00**	0.75	0.13
10	256	−0.33	**1.00**	**1.00**	**1.00**	**1.00**	**0.98**	0.39
11	2	−3.75	0.00	0.00	0.00	0.00	0.00	0.00
11	4	−1.60	0.23	0.05	0.01	0.00	0.00	0.00
11	8	−0.72	0.54	0.23	0.04	0.01	0.00	0.00
11	16	−0.46	**0.96**	0.89	0.45	0.12	0.03	0.00
11	32	−0.39	**1.00**	**1.00**	**0.91**	0.41	0.06	0.01
11	64	−0.32	**1.00**	**1.00**	**1.00**	**0.92**	0.32	0.04
11	128	−0.29	**1.00**	**1.00**	**1.00**	**1.00**	0.79	0.14
11	256	−0.27	**1.00**	**1.00**	**1.00**	**1.00**	**0.99**	0.46
12	2	−3.75	0.06	0.02	0.00	0.00	0.00	0.00
12	4	−1.58	0.22	0.03	0.00	0.00	0.00	0.00
12	8	−0.57	0.76	0.45	0.13	0.02	0.00	0.00
12	16	−0.40	**0.99**	**0.94**	0.56	0.14	0.02	0.00
12	32	−0.33	**1.00**	**1.00**	**0.96**	0.50	0.09	0.01
12	64	−0.28	**1.00**	**1.00**	**1.00**	**0.95**	0.40	0.05
12	128	−0.25	**1.00**	**1.00**	**1.00**	**1.00**	0.86	0.17
12	256	−0.24	**1.00**	**1.00**	**1.00**	**1.00**	**1.00**	0.52

The critical values given in Tables 2 and 3 can be compared to the ones given by Hillis in Table 13-1 of [14]. For the 95% confidence limit, Hillis gives the critical values −0.51, −0.45 and −0.34 for six, seven and eight taxa, and similarly −0.67, −0.60 and −0.47 for the 99% confidence limit. Hillis used *m* = 100 for the cases *n* = 6 and *n* = 7; for eight taxa, he used equal proportions of *m* = 30, *m* = 100 and *m* = 200. While his results are not directly comparable to ours due to the differing number of characters, it can be seen that the results are quite similar especially for the 99% confidence level.

Our results agree with Hillis’s observation that smaller number of characters produce more negative critical values. However, Hillis also claims that “there is very little change in the critical value for data sets with more than 100 sequence positions” (pg. 287 [14]), which seems to us to give a wrong idea about the behavior of the critical value as the number of characters increases. Comparing the critical values for 128 and 256 characters and for various values of *n*, it can be seen that the values are clearly different and no convergence has been reached. Moreover, for *n* = 5 the critical value actually appears to start decreasing again when moving from 128 to 256 characters.

Tables 2 and 3 also display how well the skewness test managed to detect the phylogenetic signal present in our simulated data. For each setting (*n*, *m*, *q*), we display the fraction of simulated data sets for which the skewness test reports the presence of phylogenetic signal. Since all the simulated data sets do by definition contain such signal, this fraction is equivalent to the correctness rate of the skewness test. Comparing the results to the success probabilities given in Table 1, it can be seen that the skewness test can detect the *presence* of phylogenetic signal even when it is unlikely that the true phylogeny can be found by considering tree lengths. In some of the cases, this distinction is striking: for instance, for *n* = 12, *m* = 256, *q* = 0.40, the skewness test detected the phylogenetic signal in 100% of the cases (rounded to the closest full percent), even though the maximum parsimony method managed to recover the true phylogeny only with probability 0.07.

It is also interesting that for six taxa and 64, 128, or 256 characters, the skewness test performs better for *q* = 0.16 than for *q* = 0.08. The general trend, however, appears to be that higher mutation probabilities in the simulated data produce data that are more often classified as not containing phylogenetic signal.

## Discussion

Our results indicate that the maximum parsimony method for inferring phylogenies performs well under a simple data-generating model if the rate of change is small enough and, crucially, if the number of characters is sufficiently large. The results complement the earlier asymptotic results by focusing on the small sample regime. For instance, if there are twelve taxa, then one should probably have at least 128 characters if one is to have any confidence that the inferred phylogeny is the true one. Moreover, since our model for simulating phylogenetic data corresponds to the no-common-mechanism model which can be shown to be the most natural model for parsimony [2], it should be expected that in more realistic settings the requirements for the number of characters are likely to be higher due to discrepancies between the underlying model and the one implied by MP. We refer the reader to [11] for further discussion about what can be inferred from simulation experiments and what are their biases.

Future work should focus on quantifying the exact relationship between the number of taxa and the number of characters required. Preferably this should be done both with the model used in this article and with more realistic models in order to enable comparisons and increase our understanding of the effect of the model on the results. This will require either more computational work or new theoretical results; the latter seems difficult due to the inherent difficulty (NP-hardness) of the problem of finding the best tree [35, 36].

Our extensive evaluation of the skewness test for detecting phylogenetic signal indicates that in all reasonable situations, the test appears to successfully detect the structure present in our simulated phylogenetic data. The test manages to detect the signal even when the data are insufficient for the maximum parsimony method to correctly infer the true phylogeny.

We have also extended the skewness test to up to twelve taxa (earlier literature went only up to eight), which is close to the limit of what the present computers are capable of handling. Our results cast doubt on the earlier claim that the critical value of the test changes little when the number of characters is increased beyond one hundred; further work is required to find out where the critical values converge as the number of characters grows.

## Supporting Information

S1 Table Simulation Results. The results of our simulations are provided in this table in the CSV format. Each row of the file corresponds to a single data set and contains the following columns:
Number of observed taxa.Number of characters.Number of possible character states. (Has always the value 4.)An indicator for whether the simulation used an unrooted binary tree for data generation (value 1) or whether the data is fully random (value 0).The mutation probability *q*, or −1 if column 4 has the value 0.Number of trees sharing the shortest length.The shortest length.The length of the true data-generating tree, or 0 if column 4 has the value 0.The minimum length of a tree *ℓ*_min_ = min_*T*_*ℓ*(*T*; *D*). (This is a duplicate of column 7.)The maximum length of a tree *ℓ*_max_ = max_*T*_*ℓ*(*T*; *D*).The rest of the columns give the number of trees having lengths *ℓ*_min_, *ℓ*_min_ + 1, ⋯, *ℓ*_max_ − 1, *ℓ*_max_. (Hence there are *ℓ*_max_ − *ℓ*_min_ + 1 of these columns.)
(ZIP)

S1 Software Package C++11 and Python Source Code. The program with which the experiments were performed is provided in a zip archive. The file contains instructions on how to compile and run the software.
(ZIP)
